# Causality of genetically proxied immunophenotypes on cardiovascular diseases: a Mendelian randomization study

**DOI:** 10.3389/fimmu.2024.1344773

**Published:** 2024-06-03

**Authors:** Xuehan Wang, Huixin Cheng, Meng Feng, Bing Jiang, Chunzhen Ren, Qilin Chen, Xiaodong Zhi, Yingdong Li

**Affiliations:** ^1^ The First Clinical Medical College, Lanzhou University, Lanzhou, Gansu, China; ^2^ Department of Emergency Medicine, Qilu Hospital of Shandong University, Jinan, Shandong, China; ^3^ School of Traditional Chinese and Western Medicine, Gansu University of Chinese Medicine, Lanzhou, Gansu, China

**Keywords:** immunity, cardiovascular diseases, causality, Mendelian randomization, genome-wide association study

## Abstract

**Background:**

Cardiovascular diseases (CVDs) stand as the foremost global cause of mortality, prompting a growing interest in using the potential of immune cells for heart injury treatment. This study aims to assess the causal association between immune cells and CVDs.

**Methods:**

A total of 731 immune cells were derived from a previously published genome-wide association study (GWAS), which included approximately 22 million genetic variants among 3,757 individuals of Sardinian ancestry. Genetic associations with atrial fibrillation (AF), heart failure, coronary artery disease, myocardial infarction and stroke were extracted from large-scale GWAS. A two-sample Mendelian randomization (MR) analysis was used to assess the causal association between immune cells and CVDs. Replication MR analysis based on FinnGen dataset and meta-analysis are sequentially conducted to validate causal relationships.

**Results:**

Collectively, genetically predicted 4 immune cell traits were associated with AF and 5 immune cell traits were associated with stroke. Increased levels of IgD- CD38dim absolute count were associated with a higher susceptibility to AF, while increased expression of CD14+ CD16+ monocytes, CD62L on CD62L+ myeloid dendritic cells, and CD16 on CD14- CD16+ monocytes were linked to a decreased susceptibility to AF. Additionally, an elevated susceptibility to stroke was linked to an increase in the percentage of CD39+ resting Tregs and heightened CD27 expression on IgD- CD38+ cells. Conversely, a decreased susceptibility to stroke was associated with increased CD40 expression on monocytes, particularly on CD14+ CD16+ and CD14+ CD16- monocytes, with the latter two showing the most compelling evidence.

**Conclusion:**

This study identified several immune cell traits that have a causal relationship with CVDs, thus confirming that immune cells play an important role in the pathogenesis of these diseases.

## 1 Introduction

Cardiovascular diseases (CVDs), such as heart failure (HF), atrial fibrillation (AF), coronary artery disease (CAD), myocardial infarction (MI) and stroke stand as the most prevalent and leading causes of mortality on a global scale. The burden of cardiovascular diseases does not derive only from the deaths it causes. The incidence and disability rates associated with CVDs are also pivotal considerations within the epidemiological landscape of these diseases (1). According to recent estimates of the Global Burden of Cardiovascular Disease spanning from 1990 to 2019, the prevalence of cardiovascular disease surged from 271 million to 523 million, with the number of cardiovascular disease-related deaths escalating from 12.1 million to 18.6 million (2). Moreover, CVDs not only engender adverse cardiovascular outcomes but also exert a deleterious influence on the emergence of dementia, thereby complicating care planning for dementia and comorbidities. In 2019, the global count of individuals afflicted by dementia reached 55 million, with projections indicating a rise to 139 million by 2050 (3). Therefore, it is of great significance to identify the relevant risk factors for the prevention and treatment of cardiovascular diseases and their complications.

The immune system occupies a pivotal role in the development and homeostasis of the human body. A mounting body of evidence underscores the involvement of immune cells in the pathogenesis and progression of various diseases, involving nervous system diseases, endocrine and metabolic diseases and cancer (4–6). In recent years, the substantial contribution of the immune system to the normal functioning of the heart and its response to injury has emerged as a compelling area of research (7). The precise modulation of the immune system to promote myocardial recovery and repair is an important goal of cardio-immunology. Mechanisms of immune effects in the cardiovascular system include the activation and transportation of immune cells to the heart. Infiltration of immune cells within the myocardium may have adverse implications for the heart and contribute to the development of CVDs (8). However, the relationship between the immune cells and CVDs is intricate and unclear.

Mendelian randomization (MR) analysis represents a method that emulates randomized controlled trials by using genetic variables to assess the causal relationships between exposures and clinical outcomes of diseases. This method of analysis can avoid the influence of confounding factors and reverse causation (9), rendering it a viable means to explore the causal association between immune cell traits and CVDs. Therefore, we conducted a two-sample MR analysis by using summary statistics from large scale genome-wide association studies (GWAS) of immune cells, HF, AF, CAD, MI and stroke to evaluate the causal relationship between immune cell traits and cardiovascular diseases.

## 2 Methods

### 2.1 Study overview

**Figure 1:**
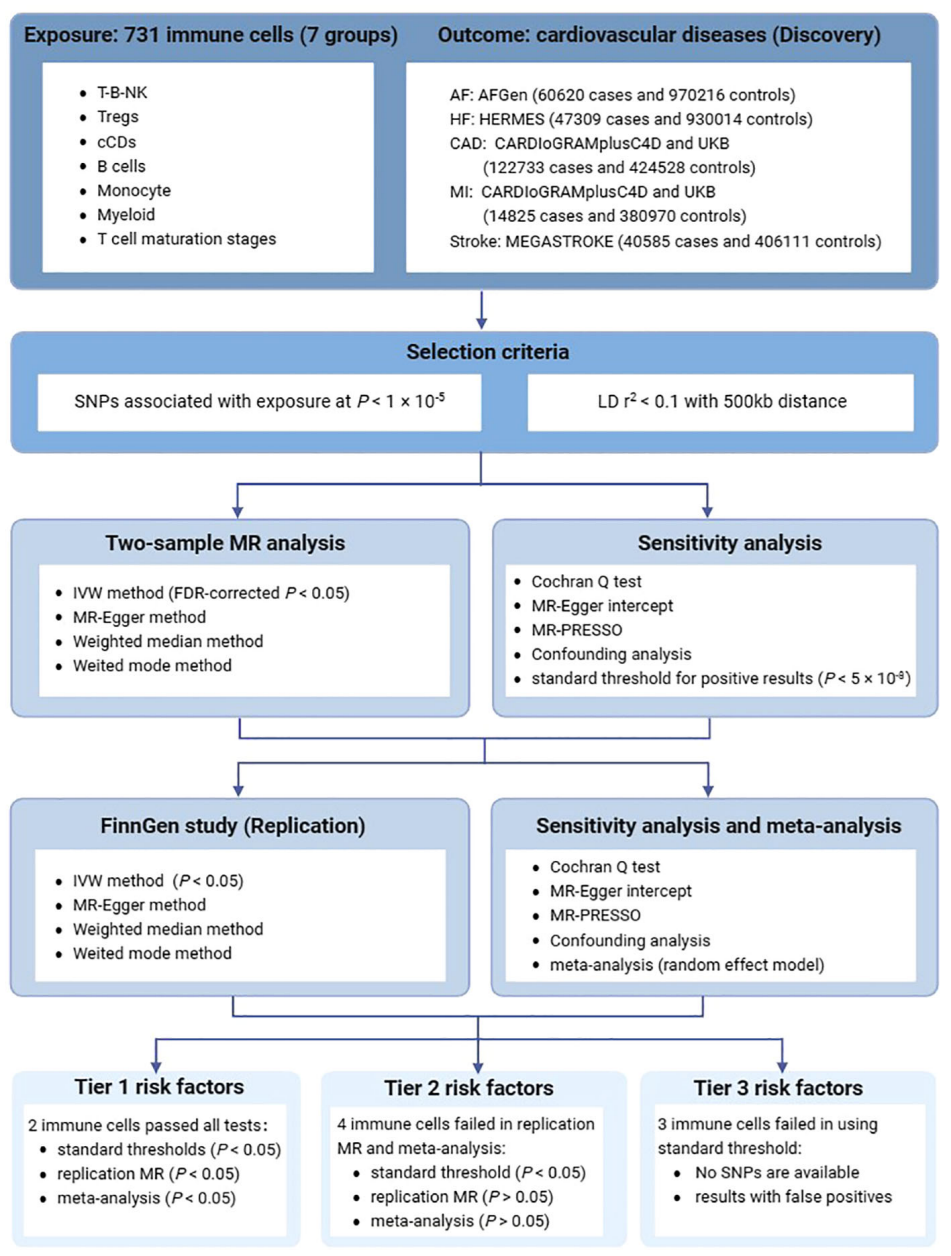
An overview of the study design. AF, atrial fibrillation; HF, heart failure; CAD, Coronary artery disease; MI, Myocardial infarction; SNPs, single nucleotide polymorphisms; LD, linkage disequilibrium; MR, Mendelian randomization; IVW, inverse variance weighted method.

In this study, each immune cell trait was treated as an independent exposure variable. A two-sample MR design was performed to assess the potential causal association between immune cell traits and cardiovascular diseases. The validity of MR analysis relies on the satisfaction of three crucial assumptions (10). First, the genetic variants (IVs) must directly affect the exposure. Second, IVs should not exhibit associations with any known confounding factors. Third, IVs must influence the risk of the outcomes solely through their impact on the exposure. An overview of this two-sample MR design is presented in **Figure 1**.

### 2.2 GWAS data for immune cells

Our data on immune cells were drawn from genome-wide association studies (GWAS) encompassing approximately 22 million genetic variants within a cohort of 3,757 individuals of Sardinian descent (11). These GWAS data encompassed a comprehensive set of 731 immune cell traits, which included 118 absolute cell (AC) counts, 192 relative counts, 389 median fluorescence intensities (MFIs) of surface antigens, and 32 morphological parameters. These traits could be categorized into 7 panels: T-B-NK, Tregs, conventional dendritic cells (cDCs), B cells, Monocytes, Myeloid cells, and T cell maturation stages. The complete GWAS summary statistics are accessible in the GWAS Catalog, with accession numbers ranging from GCST0001391 to GCST0002121.

### 2.3 GWAS data for cardiovascular disease

We selected AF, HF, CAD, MI and all-cause stroke as the cardiovascular disease outcomes in our study. To minimize potential biases arising from population heterogeneity, the study populations in most research have predominantly comprised individuals of European ancestry (12–16). The GWAS summary statistics for AF were obtained from Atrial Fibrillation Consortium (AFGen) Consortium. The AFGen Consortium conducted a meta-analysis of five cohort studies, involving a total of 60,620 cases and 970,216 controls. The single nucleotide polymorphisms (SNPs) associated with HF were derived from Heart Failure Molecular Epidemiology for Therapeutic Targets consortium (HERMES), including 47309 cases and 930014 controls. Summary-level statistical data for CAD and MI were abstracted from UK Biobank and meta-analyses with Coronary Artery Disease Genome-wide Replication and Meta-Analysis plus The Coronary Artery Disease (CARDIoGRAMplusC4D) 1,000 genome-based GWAS data, including 547261 participants (122733 cases and 424528 controls) for CAD and 395795 participants (14825 cases and 380970 controls) for MI. It is worth noting that the 77% of the GWAS data for CAD and MI in the CARDIoGRAMplusC4D consortium were of European descent. Summary statistics for stroke were acquired from the Multiancestry Genome‐wide Association Study of Stroke (MEGASTROKE) consortium, which involved 40585 cases and 406111 controls. Two GWAS summary data included AF (50743 cases and 210652 controls) and stroke (43132 cases and 297867 controls) of European ancestry from Finnish GWAS datasets Release 10 (FinnGen R10) were employed in the replication MR (https://r10.finngen.fi/). There was no evidence of sample overlap across these GWASs. Detailed information regarding the cardiovascular diseases considered in this study is provided in **Supplementary Table 1**.

### 2.4 Genetic variants selection criteria

We set the significance level for individual immune cells at *P* < 1×10^-5^, considering linkage disequilibrium (LD) with r^2^ < 0.1 and an LD distance greater than 500kb, given the limited number of available SNPs (17–19). The reference panel used in this study was the European population from the 1000 Genomes Phase 3 (20). If a sufficient number of SNPs are available, repeat the analysis using the standard threshold (*P* < 5×10^-8^) for positive results. For cardiovascular diseases, the significance level for IVs was set *P* < 5×10^-8^, with LD criteria of r^2^ < 0.001 and an LD distance exceeding 10,000kb. Considering the limited impact of a small amount of missing data on the results, some missing IVs have not been replaced by proxy SNPs. Furthermore, SNPs were removed during the harmonization process due to palindromic with intermediate allele frequency or incompatible. We used the following formula to calculate the F-statistic: F = R^2^ × (N - k - 1)/[(1 – R^2^) × k, where N represents the sample size of the exposure, k denotes the number of SNPs, and R^2^ refers to the proportion of variance in the exposure variable that is explained by the genetic instruments used in the analysis. In addition, R^2^ was calculated using 2 × (1 - EAF) × EAF × beta^2^, where EAF represents the effect allele frequency, and beta denotes the estimated genetic effect of each SNP on the exposure (21). A threshold of F > 10 indicating a relatively strong estimation effect in the MR analyses.

### 2.5 Statistical analysis

Causal associations between immune cells and CVDs in the MR analysis were primarily assessed by using the standard inverse variance weighted (IVW) method (22). Heterogeneity was assessed using the Cochran Q test (23), and if no heterogeneity was detected (*P* > 0.05), IVW analysis was performed using a fixed-effects model; otherwise, a random-effects model was applied. Supplementary analyses included the MR-Egger method, Weighted Mode method, and Weighted Median method (24–26). MR Pleiotropy Residual Sum and Outlier (MR-PRESSO) was employed to identify potential outliers, while both the MR-Egger intercept test and the MR-PRESSO global test were utilized to identify horizontal pleiotropy (24, 27). If needed, addressing heterogeneity involved the removal of outliers, and adjustments for pleiotropy were made by adopting a higher significance threshold (*P* < 5×10^-8^) to select genetic variants. We used a phenome-wide association test (28) (PhenoScanner V2: http://www.phenoscanner.medschl.cam.ac.uk) to examine the correlation between selected SNPs and potential confounders, primarily focusing on body mass index, alcohol intake, dyslipidemia, hypertension, diabetes, atrial fibrillation, coronary artery disease, venous embolism and thrombosis. A statistically significant association is established when the False Discovery Rate (FDR) for the estimated causal effect of a particular immune cell is less than 0.05 (29). An online power calculation tool (https://shiny.cnsgenomics.com/mRnd/) was used to evaluate the statistical power of causal effect estimates, considering a power threshold of 0.8 as appropriate (30). Subsequently, if a sufficient number of SNPs are available, we conducted a repeat analysis on the positive results using standard threshold (*P* < 5×10^-8^) to determine whether they were still significant. Scatter and funnel plots were employed to detect outliers and pleiotropy. In order to avoid the influence of single SNP on the results and ensure the stability of MR Analysis results, the leave-one-out sensitivity analysis was performed. Based on the GWAS summary data from FinnGen, further replication MR analysis (using standard threshold) was conducted on the positive findings. Finally, estimates from the discovery study and FinnGen were combined using the random-effects meta-analysis method.

All analyses were performed using RStudio (R version 4.3.1). TwoSampleMR package (version 0.5.10) and MRPRESSO package (version 1.0) were used to perform data analysis for the MR study in this research. Data visualization was performed using the forestploter package.

## 3 Results

### 3.1 The preliminary analysis results for the relationship between immune cell traits and cardiovascular diseases using 1×10^-5^ threshold

**Figure 2:**
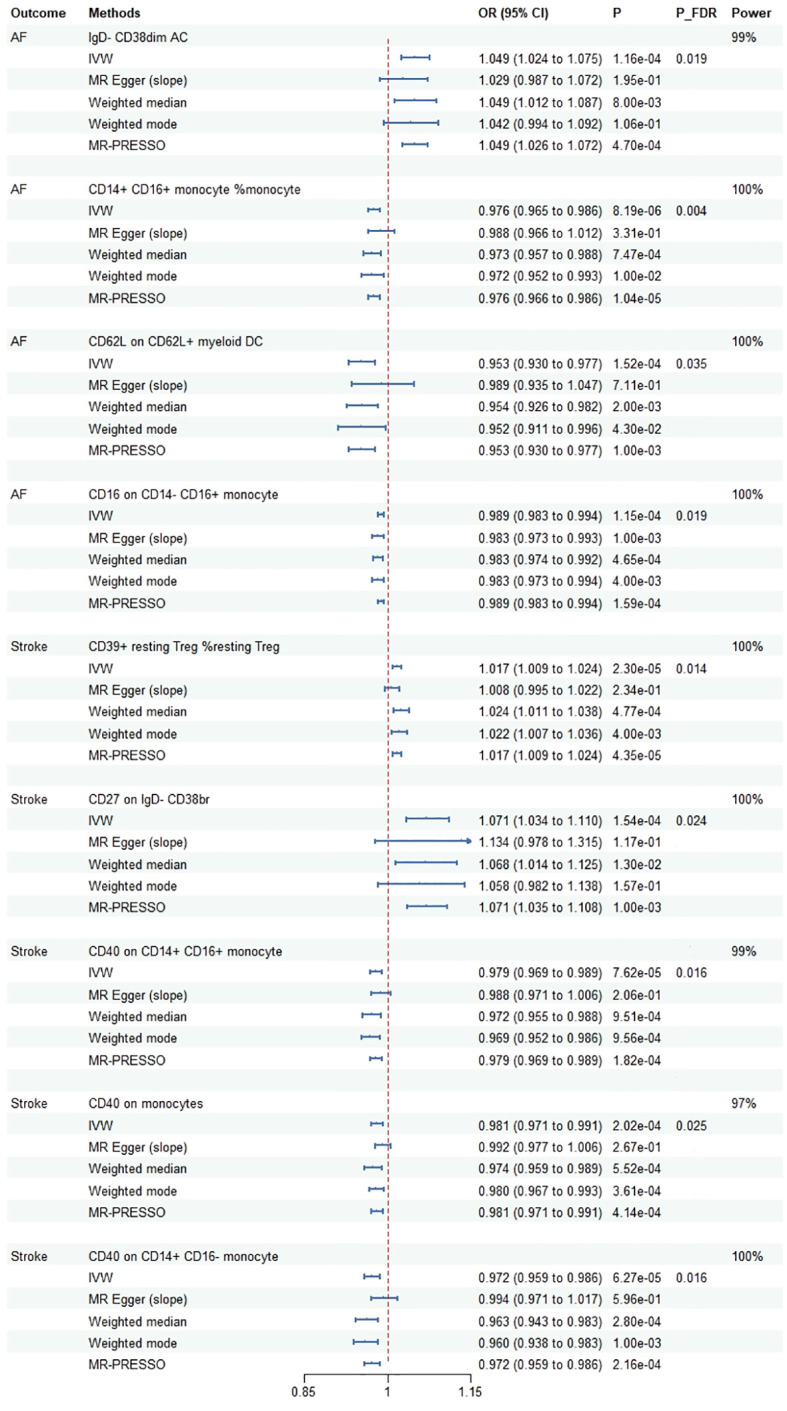
Mendelian randomization estimates for the causal effect of immune cell traits on the risk of cardiovascular diseases passing false discovery rate (FDR) correction. AF, atrial fibrillation; OR, odds ratio; CI, confidence interval; IVW, inverse variance weighted method; FDR, false discovery rate; MR-PRESSO, MR Pleiotropy Residual Sum and Outlier.

The details of the IVs are shown in **Supplementary Table 2**. In this MR analysis, a total of 307 suggestive association were identified (*P* < 0.05) (**Supplementary Table 3**). We observed 9 causal associations with multiple-testing corrected significance by using the IVW method (*P*_(FDR)_ < 0.05), involving 9 immune traits, which 5 were in the Monocyte panel, 2 in the B cell panel, 1 in the cDC panel and 1 in the Treg panel (**Figure 2**). For the causal effect of cardiovascular diseases on immune cell traits, only one suggestive association was identified in the relationship between CAD and immune cell traits [odds ratio (OR): 1.155, 95% confidence interval (CI): 1.000–1.333, *P* = 0.049]. However, there was no statistical difference after multiple-testing corrected (*P*_(FDR)_ = 0.999) (**Supplementary Table 4**).

Cochrane’s Q test showed that only in CD62L on CD62L+ myeloid DC did significant heterogeneity exist with AF (*P* = 0.018). One outlier (rs12755775) was identified using MR-PRESSO and then removed further analysis. There was no evidence of pleiotropy existing for immune cell traits according to MR-PRESSO global test and MR-Egger intercept except for the correlation between CD40 on CD14+ CD16- monocyte and stroke (*P* = 0.024). Specific information on the sensitivity analysis is shown in the **Supplementary Table 3**. Furthermore, SNPs related to AF and stroke were searching from the PhenoScanner database and 15 SNPs were excluded (rs1027700: type 2 diabetes, rs17456856: aortic valve disorder, rs73025536: myocarditis, rs76669856: venous embolism and thrombosis, rs10882140: high cholesterol, rs11187826: hypertension, rs147121076: hypertensive heart disease with heart failure, rs17108042: coronary artery disease, rs2648723: body mass index, rs7080472: hypertension, rs7896439: hypertension, rs16991213: alcohol intake, rs1801274: total cholesterol, rs78648967: alcohol intake, rs77869433: atrial fibrillation and flutter). After excluding outlier and potential confound-associated SNPs, we founded that the corrected results had a similar statistical value and direction with raw analysis (**Supplementary Table 5**).

### 3.2 MR analysis for significant results using the standard threshold

Subsequently, we conducted a repeat analysis on the positive results using standard thresholds (*P* < 5×10^-8^). The results were as follows: CD14+ CD16+ monocyte (%monocyte) (OR: 0.965, 95% CI: 0.951–0.979, *P* = 1.43×10^-6^], and CD16 on CD14- CD16+ monocyte (OR: 0.988, 95%CI: 0.981–0.996, *P* = 0.002) were identified to have negative causal effects on AF. Although the estimated value of the IVW method is significant for CD62L on CD62L+ myeloid dendritic cells (OR: 0.928, 95%CI: 0.900–0.956, *P* = 1.15×10^-6^), there is inconsistency in the beta direction in the MR-Egger method. In addition, CD40 on CD14+ CD16+ monocyte (OR: 0.960, 95%CI: 0.945–0.976, *P* = 1.12×10^-6^), CD40 on monocytes (OR: 0.969, 95%CI: 0.951–0.986, *P* = 5.15×10^-4^) and CD40 on CD14+ CD16- monocyte (OR: 0.958, 95%CI: 0.941–0.976, *P* = 3.88×10^-6^) were identified to have negative causal effects on stroke, while CD39+ resting Treg %resting Treg (OR: 1.017, 95%CI: 1.009–1.025, *P* = 5.66×10^-5^) had positive causal effect on stroke (**Supplementary Table 6**).

Sensitivity analysis showed that Cochran’s Q-derived P-values were all greater than 0.05. All MR-Egger intercept tests yielded P-values higher than 0.05, indicating the absence of significant horizontal pleiotropy. The resulting visualization of the scatter plots and funnel plot were shown in **Supplementary Figure 1**, **Supplementary Figure 2**. Leave-one-out analyses indicated that causal estimates were minimally affected after excluding any individual SNP (**Supplementary Figure 3**).

### 3.3 Results of replication MR analysis and meta-analysis

**Figure 3:**
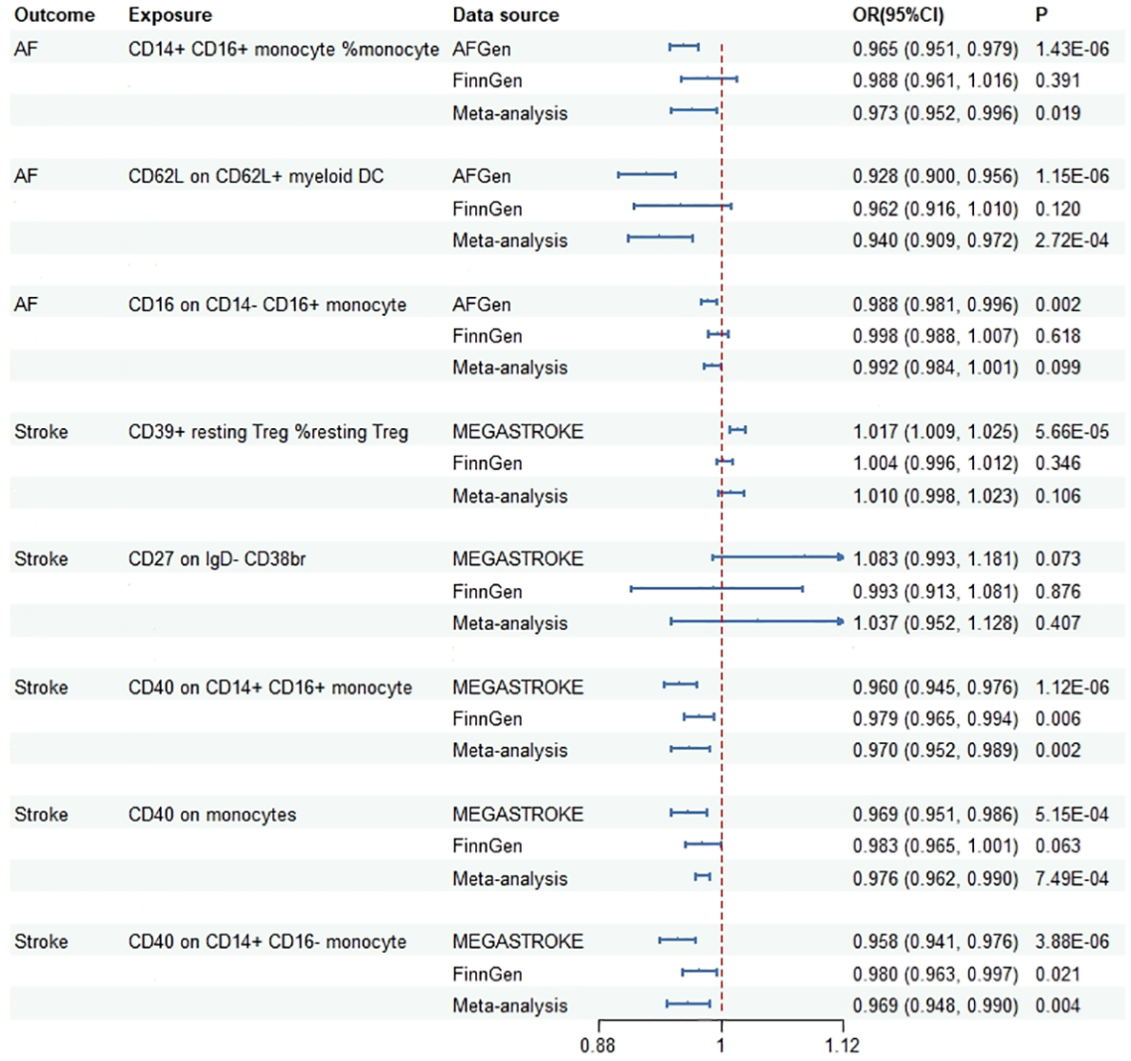
Meta-analysis results of the association between genetically predicted immune cells and risk of cardiovascular diseases based on Mendelian randomization analysis using standard threshold. AF, atrial fibrillation; OR, odds ratio; CI, confidence interval.

During the replication stage, two immune cells were successfully confirmed in the FinnGen dataset using the IVW method (**Supplementary Table 6**). Scatter plots, funnel plot and leave-one-out analyses were shown in **Supplementary Figure 4**, **Supplementary Figure 5**, **Supplementary Figure 6**. In the meta-analysis of discovery and replication study, five immune cell traits showed significant associations (**Figure 3**). The OR (95%CI) of AF per standard deviation (SD) increase in genetically predicted immune cells was 0.973 (0.952, 0.006) for CD14+ CD16+ monocyte (%monocyte), 0.940 (0.909, 0.972) for CD62L on CD62L+ myeloid DC. In addition, the OR (95%CI) of stroke per SD increase in genetically predicted immune cells was 0.970 (0.952, 0.989) for CD40 on CD14+ CD16+ monocyte, 0.976 (0.962, 0.990) for CD40 on monocyte and 0.969 (0.948, 0.990) for CD40 on CD14+ CD16- monocyte.

Based on the evidence presented, we have categorized the characteristics of these immune cells into three tiers. Two immune cell types (CD40 on CD14+ CD16+ monocyte, CD40 on CD14+ CD16- monocyte) passed all the tests and were classified as Tier 1, indicating a strong correlation. Four immune cells (CD14+ CD16+ monocyte %monocyte, CD16 on CD14- CD16+ monocyte, CD39+ resting Treg %resting Treg, CD40 on monocytes) failed in the replication MR analysis or the meta-analysis, and were classified as Tier 2. Three immune cells (IgD- CD38dim AC, CD62L on CD62L+ myeloid DC, CD27 on IgD- CD38br) either had an insufficient number of available SNPs when using standard thresholds, or the results exhibited potential false positives, and were classified as Tier 3.

## 4 Discussion

Although the immune system plays an important role in the pathogenesis of CVDs, the precise causal relationship between immune cell traits and CVDs remains unclear. This is the first MR analysis to assess whether immune cells exert a causal influence on the risk of CVDs, as well as whether an inverse association exists. In total, we identified 237 immune cell traits that exhibited a causal effect on CVDs, and among them, nine immune cell types remained statistically significant even after applying appropriate statistical corrections. The use of standard thresholds, replication of MR analysis, and meta-analysis were employed to validate the causal relationship between immune cell traits and cardiovascular diseases. In summary, we found two types of immune cells (CD40 on CD14+ CD16+ monocyte, CD40 on CD14+ CD16- monocyte) with the most compelling evidence (Tier 1), four types of immune cells (CD14+ CD16+ monocyte %monocyte, CD16 on CD14- CD16+ monocyte, CD39+ resting Treg %resting Treg, CD40 on monocytes) with moderately compelling evidence (Tier 2), and three types of immune cells (IgD- CD38dim AC, CD62L on CD62L+ myeloid DC, CD27 on IgD- CD38br) with suggestive evidence (Tier 3). For the causal effect of immune cell traits on cardiovascular diseases, only one suggestive association was identified in the relationship between immune cell traits and CAD. Notably, this association did not achieve statistical significance after correction.

Our research indicates that the decreased susceptibility to stroke is associated with an increase in the expression of CD40 in monocytes, particularly in CD14+ CD16+ and CD14+ CD16- monocytes, with the latter two demonstrating the most compelling evidence. CD40 is a type I transmembrane glycoprotein receptor expressed on various immune cell types, including B cells, monocytes, endothelial cells, vascular smooth muscle cells and fibroblasts [52].CD40, along with its ligand CD40L, plays crucial roles in various aspects of the immune system. The effects of CD40 and CD40L on atherosclerosis, specifically atherothrombosis, have been well studied (31). As a well-recognized marker of atherosclerosis inflammation, elevated CD40L was found to be associated with worse clinical outcome following stroke (32). Conversely, another study showed that CD40L was associated with markers of inflammation, it did not independently serve as a risk marker for stroke (33). In addition, severe immunosuppression and unexpected thromboembolic events have been reported in preclinical and clinical trials involving anti-CD40L (34, 35). However, these observational associations are insufficient to establish a causal relationship between CD40 and stroke. In our study, elevated CD40 expression may be associated with decreased susceptibility to stroke. This is consistent with the findings of several Mendelian randomization studies, which have shown that CD40 is associated with a reduce risk of aortic disease, enhanced cognitive function, and a decreased risk of stroke. In previous studies, most attention has focused on CD40L and the role of CD40/CD40L in promoting disease progression. Although CD40L acts as a ligand of CD40, they may have distinct roles in disease pathogenesis, and the role of CD40 and CD40L in thrombus stabilization may be a double-edged sword.

Our research findings demonstrate that the expression of CD14 and CD16 contributes to the improvement of AF risk. Human monocytes exhibit immunophenotype heterogeneity based on the expression of lipopolysaccharide receptors CD14 and CD16. CD14++ CD16− monocytes, often referred to as classical monocytes, constitute the main subtype circulating in the blood of healthy individuals, comprising approximately 85% of all monocytes. In contrast, CD14+ CD16+ monocytes, categorized as intermediate monocytes or non-classical monocytes, are relatively rare. Non-classical monocytes are widely believed to possess anti-inflammatory effects, as they are capable of maintaining vascular homeostasis (36). Suzuki et al. (37) have identified an elevated presence of CD14++ CD16+ monocytes in patients with AF, with a likely underlying mechanism linked to the inflammatory response incited by AF. Notably, CD14+ CD16+ monocytes may possess distinct phagocytic and antigen presentation capabilities when compared to CD14++ monocytes (38). Although there is an increase in the number of CD14+ CD16+ monocytes during states of inflammation, the recruitment of circulating immune cells is thought to be a repair response after myocardial injury (39). Frauke et al. (39) confirmed that the number of CD14+ was negatively associated with the presence of cardiac fibrosis. In addition, previous researches have demonstrated a negative correlation between CD16− monocytes and the degree of recovery of left ventricular ejection fraction following acute myocardial infarction. This suggests that an increase in the number or activity of CD16− monocytes may have an adverse effect on heart disease, while the opposite was observed in CD16+ monocytes (40, 41). Our study revealed that CD14+ and CD16+ has a protective effect against AF rather than after the onset of disease, and the underlying mechanism may be due to the increased myocardial protection.

In addition, there is moderately compelling evidence of the impact of CD39+ resting Treg on the risk of stroke. CD39, an extracellular nucleotidase, is expressed on B cells, activated NK cell subsets, T lymphocytes, and endothelial cells (42). The purinergic signaling system (including ATP, ADP, AMP and ADO) centered on CD39 plays an important role in regulating vascular homeostasis and response to vascular injury. ATP and ADP are converted to AMP by CD39, and then CD73 dephosphorylates AMP into adenosine, which itself has anti-inflammatory and antithrombotic signaling properties (43). Research has demonstrated that Mice overexpressing CD39 exhibited an extended duration of arterial thrombosis, while CD73-/- mice showed a shortened period for ferric chloride-induced arterial thrombosis (44). In the context of venous thrombosis, the absence of CD39 leads to an increase in venous thrombosis due to heightened recruitment of leukocytes and the release of interleukin-1β (45). Baek et al. (46) established a cerebral ischemia model using transgenic mice expressing human CD39. The results showed that CD39 transgenic mice exhibited reduced leukocyte infiltration, smaller infarct volumes, and less nerve damage compared to wild-type mice. These findings suggest that CD39 plays an important role not only in inhibiting thrombosis, but also in reducing neuroinflammation. A recent study has found that CD39 emerges as a key immunomodulatory component for neuroprotection in ischemic stroke on CD39+ activated CD4 Treg cells (47). Similar to our results, an elevated proportion of CD39+ resting T cells, signifying a reduced proportion of activated CD39+ T cells, exerts an adverse influence on stroke. Previous researches have established that targeted CD39 therapy presents a promising therapeutic avenue for stroke management (48, 49). These observational studies hold promise for the intervention of CD39 in Treg therapy for stroke treatment.

The strength of this study lies in our systematic investigation of the relationship between immune cell characteristics and the risk of CVDs. We employed a two-sample MR design, which offers the advantages of a large sample size, broad coverage of immune cell types, reduced risk of reverse causation and confounding bias. Furthermore, the consistency of research findings across multiple analytical approaches, such as using different thresholds, replication MR analysis and meta-analysis, confirms the robustness of the research results. Nevertheless, this study has several limitations. Firstly, the study populations in most research have predominantly comprised individuals of European ancestry, and our results may not apply to non-European populations due potential ethnic disparities. Secondly, the influence of gender and age on cardiovascular disease risk is of paramount importance; however, we were unable to stratify by gender and age due to the absence of relevant GWAS data. Thirdly, our investigation primarily focuses on analyzing immune cell phenotypes in peripheral blood. Further studies to analyze immune cell traits in cardiac tissue will help identify more promising relevant biomarkers, intervention targets and prognostic indicators.

## 5 Conclusion

In summary, this MR analysis has examined the causal relationship between immune cell traits and five types of cardiovascular diseases. Causal associations between four immune cell traits and AF as well as five immune cell traits and stroke were finally identified. These findings provide preliminary evidence regarding the impact of genetically proxied immunophenotypes on the risk of cardiovascular diseases, with potential implications for future drug target selection. Further studies are required to investigate the underlying mechanisms of these causal relationships.

## Data availability statement

The original contributions presented in the study are included in the article/**Supplementary Material**. Further inquiries can be directed to the corresponding author/s.

## Author contributions

XW: Data curation, Methodology, Software, Writing – original draft. HC: Data curation, Methodology, Writing – review & editing. MF: Data curation, Resources, Validation, Writing – review & editing. BJ: Formal Analysis, Visualization, Writing – review & editing. CR: Data curation, Validation, Writing – review & editing. QC: Formal Analysis, Resources, Writing – review & editing. XZ: Data curation, Supervision, Writing – review & editing. YL: Funding acquisition, Supervision, Writing – review & editing.
