# Supplementary material for: Causality of genetically proxied immunophenotypes on cardiovascular diseases: a Mendelian randomization study
Source: Front Immunol. 2024 Jun 3;15:1344773. doi: 10.3389/fimmu.2024.1344773 (PMC11181691; doi:10.3389/fimmu.2024.1344773)
Supplement: Supplementary file 2 [file DataSheet_2.docx]

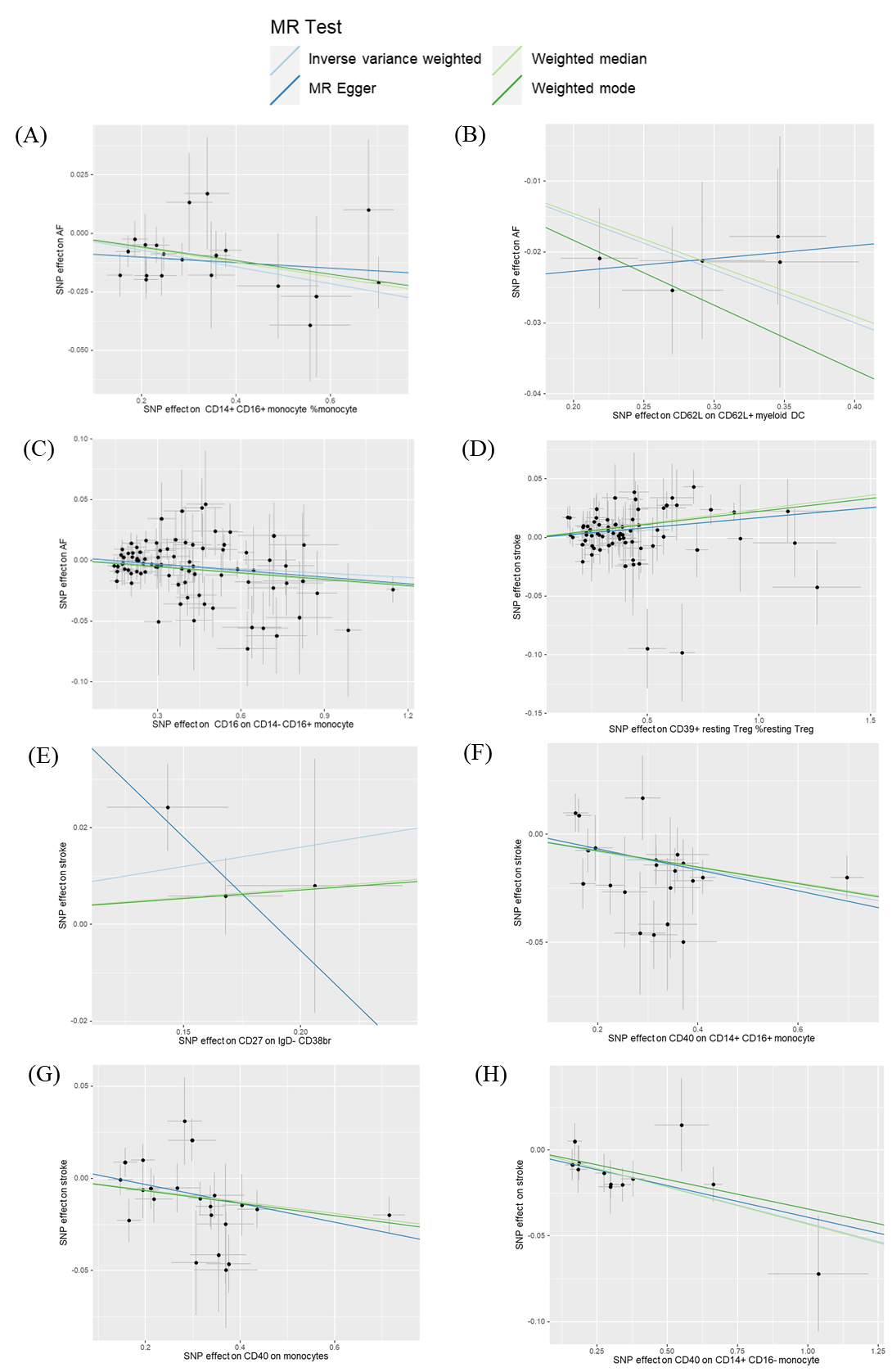


**Supplementary Figure 1.** Scatter plot of Mendelian randomization analyses of the association of immune cell traits with the risk of CVDs based on AFGen and MEGASTROKE datasets. (A) CD14+ CD16+ monocyte %monocyte on AF, (B) CD62L on CD62L+ myeloid DC on AF, (C) CD16 on CD14- CD16+ monocyte on AF, (D) CD39+ resting Treg %resting Treg on stroke, (E) CD27 on IgD- CD38br on stroke, (F) CD40 on CD14+ CD16+ monocyte on stroke, (G) CD40 on monocytes on stroke, (H) CD40 on CD14+ CD16- monocyte on stroke


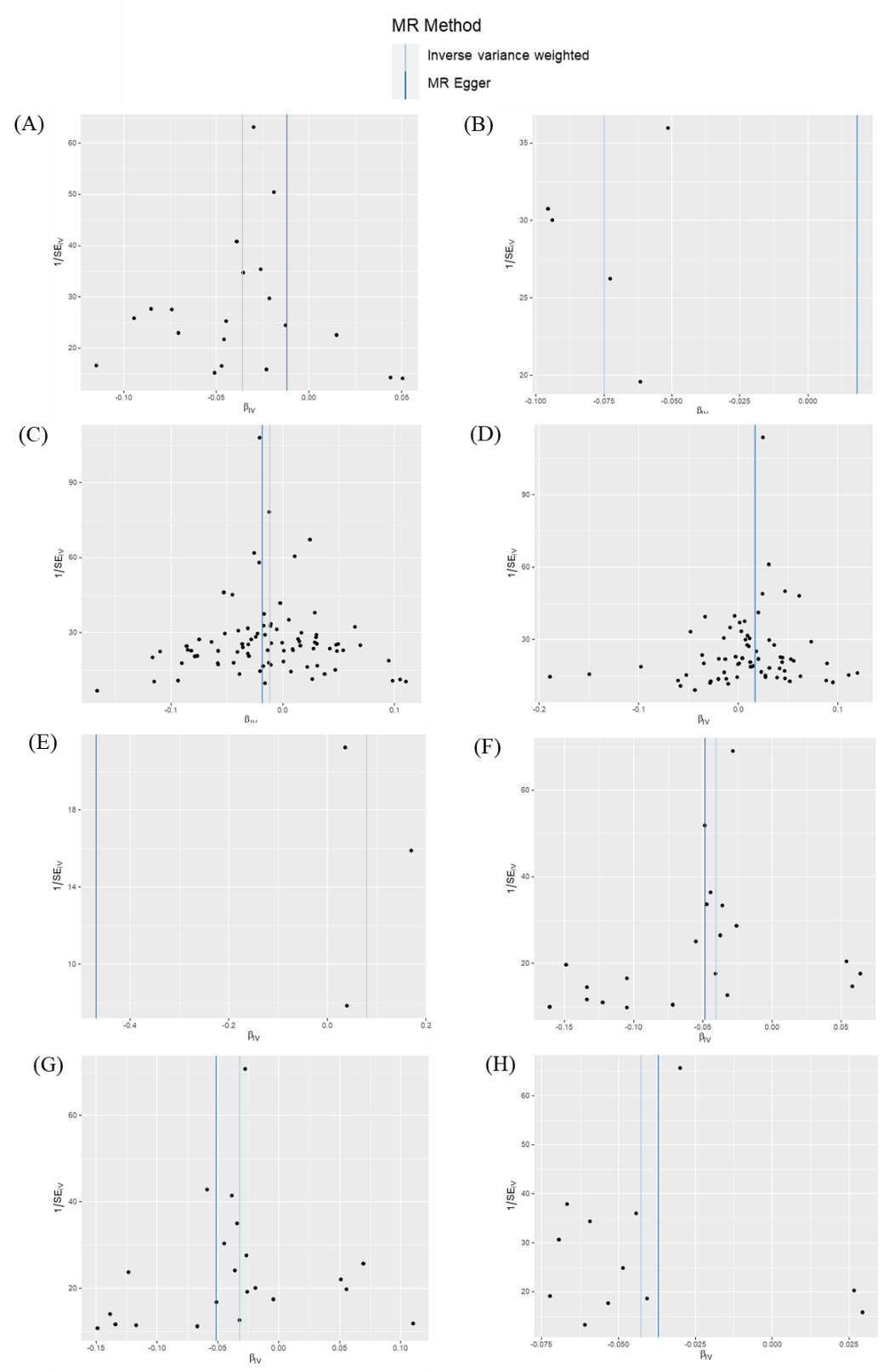


**Supplementary Figure 2.** Funnel plot represents SNPs for each significant causal association between immune cell traits and CVDs (GWAS data were from AFGen and MEGASTROKE). (A) CD14+ CD16+ monocyte %monocyte on AF, (B) CD62L on CD62L+ myeloid DC on AF, (C) CD16 on CD14- CD16+ monocyte on AF, (D) CD39+ resting Treg %resting Treg on stroke, (E) CD27 on IgD- CD38br on stroke, (F) CD40 on CD14+ CD16+ monocyte on stroke, (G) CD40 on monocytes on stroke, (H) CD40 on CD14+ CD16- monocyte on stroke


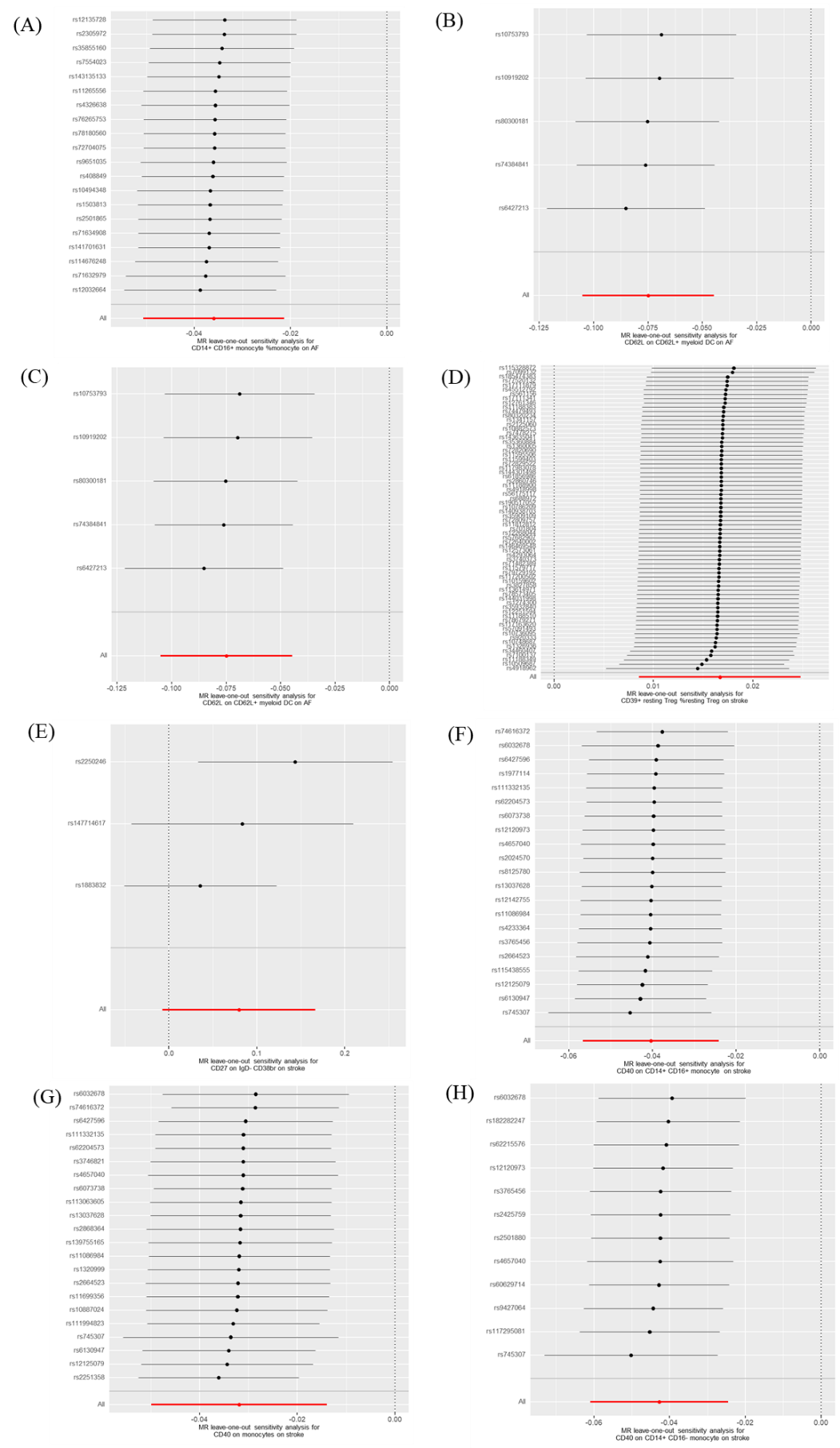


**Supplementary Figure 3.** Plots of leave-one-out analyses for Mendelian randomization analysis (GWAS data were from AFGen and MEGASTROKE). (A) CD14+ CD16+ monocyte %monocyte on AF, (B) CD62L on CD62L+ myeloid DC on AF, (C) CD16 on CD14- CD16+ monocyte on AF, (D) CD39+ resting Treg %resting Treg on stroke, (E) CD27 on IgD- CD38br on stroke, (F) CD40 on CD14+ CD16+ monocyte on stroke, (G) CD40 on monocytes on stroke, (H) CD40 on CD14+ CD16- monocyte on stroke


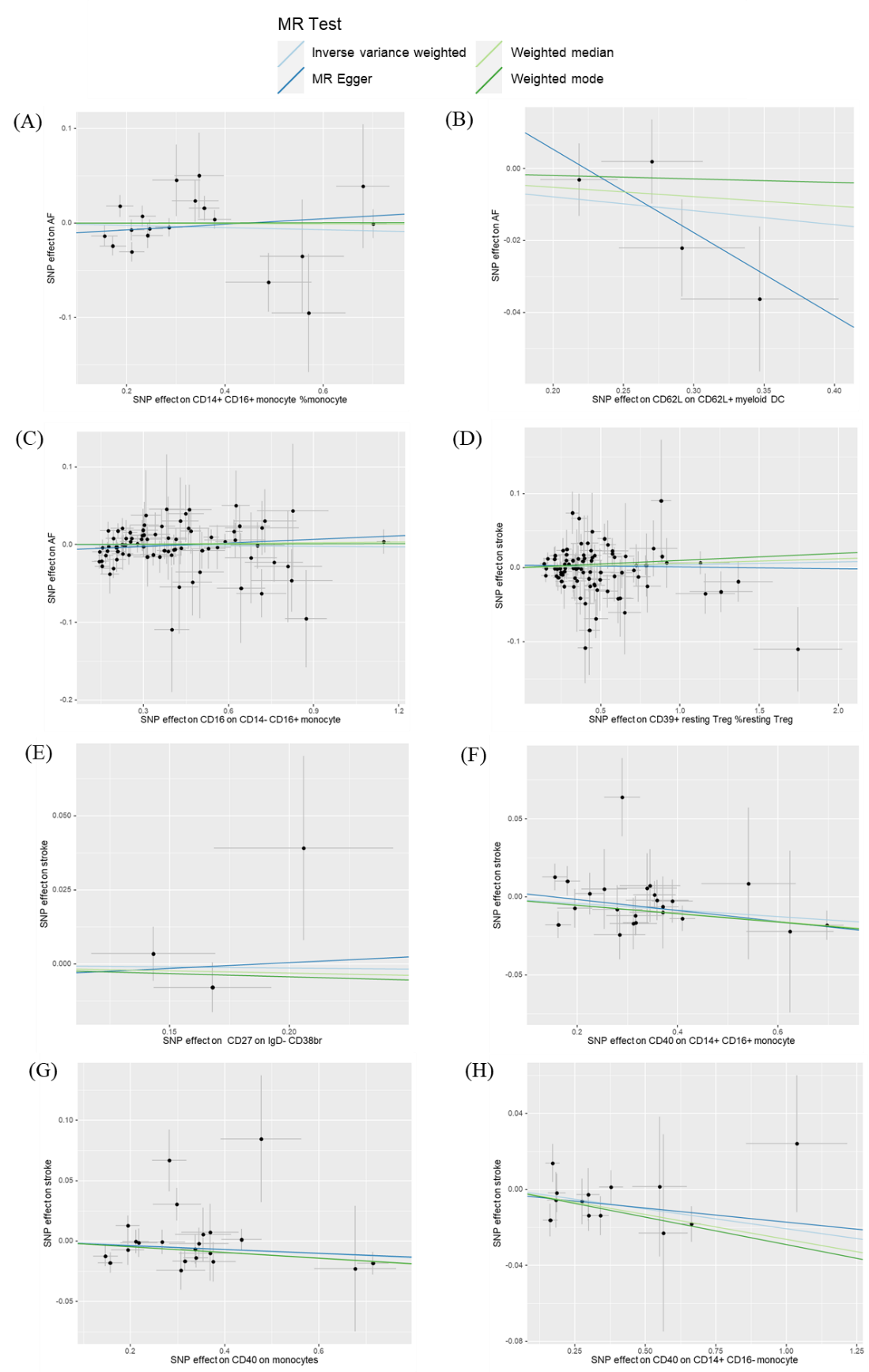


**Supplementary Figure 4.** Scatter plot of Mendelian randomization analyses of the association of immune cell traits with the risk of CVDs based on FinnGen dataset. (A) CD14+ CD16+ monocyte %monocyte on AF, (B) CD62L on CD62L+ myeloid DC on AF, (C) CD16 on CD14- CD16+ monocyte on AF, (D) CD39+ resting Treg %resting Treg on stroke, (E) CD27 on IgD- CD38br on stroke, (F) CD40 on CD14+ CD16+ monocyte on stroke, (G) CD40 on monocytes on stroke, (H) CD40 on CD14+ CD16- monocyte on stroke


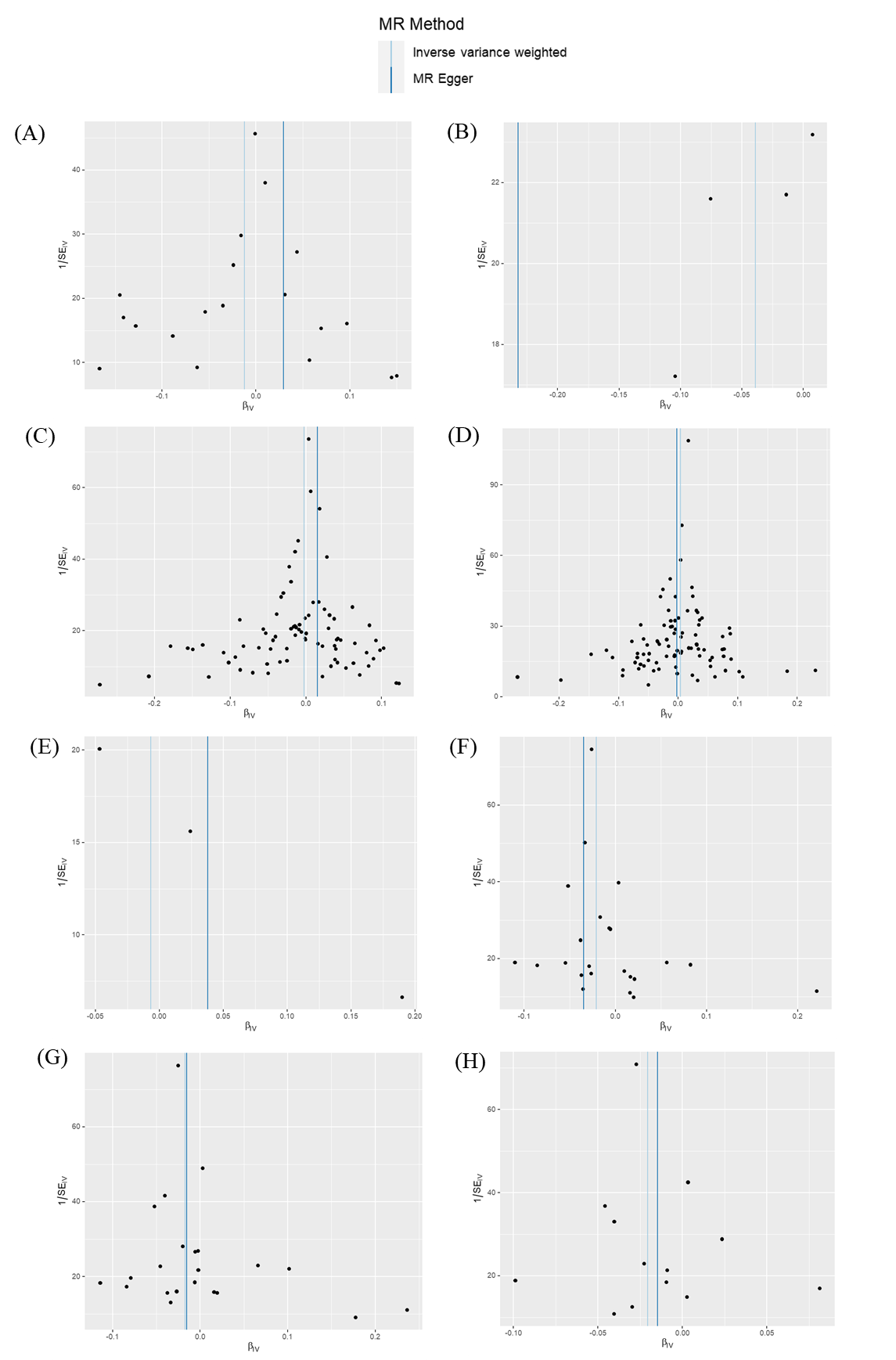


**Supplementary Figure 5.** Funnel plot represents SNPs for each significant causal association between immune cell traits and CVDs (GWAS data were from FinnGen). (A) CD14+ CD16+ monocyte %monocyte on AF, (B) CD62L on CD62L+ myeloid DC on AF, (C) CD16 on CD14- CD16+ monocyte on AF, (D) CD39+ resting Treg %resting Treg on stroke, (E) CD27 on IgD- CD38br on stroke, (F) CD40 on CD14+ CD16+ monocyte on stroke, (G) CD40 on monocytes on stroke, (H) CD40 on CD14+ CD16- monocyte on stroke


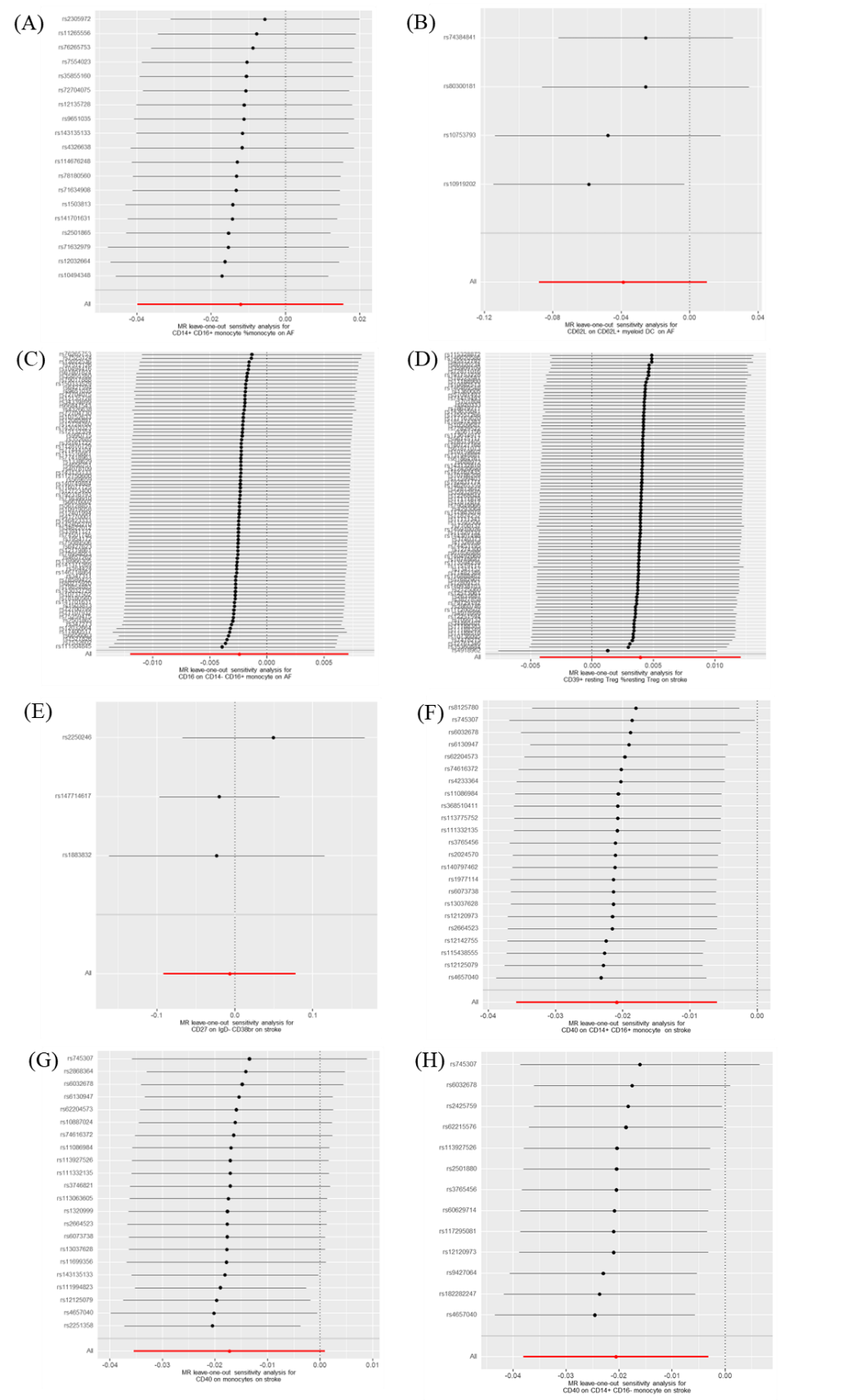


**Supplementary Figure 6.** Plots of leave-one-out analyses for Mendelian randomization analysis (GWAS data were from FinnGen). (A) CD14+ CD16+ monocyte %monocyte on AF, (B) CD62L on CD62L+ myeloid DC on AF, (C) CD16 on CD14- CD16+ monocyte on AF, (D) CD39+ resting Treg %resting Treg on stroke, (E) CD27 on IgD- CD38br on stroke, (F) CD40 on CD14+ CD16+ monocyte on stroke, (G) CD40 on monocytes on stroke, (H) CD40 on CD14+ CD16- monocyte on stroke
